# Long-Term Epidemiological Trends and Risk Factors of Crimean–Congo Hemorrhagic Fever in Kazakhstan: An Integrated Epidemiological and Entomological Analysis

**DOI:** 10.3390/v18080840

**Published:** 2026-07-31

**Authors:** Gulzhan N. Abuova, Yerkin B. Bukharbayev, Farida A. Berdaliyeva, Natalia Yu. Pshenichnaya, Bekzhan Ye. Bektan

**Affiliations:** 1 Department of Infectious Diseases, South-Kazakhstan Medical Academy, 160016 Shymkent, Kazakhstan; kz_erkin@mail.ru (Y.B.B.); fberdalieva@mail.ru (F.A.B.); bektanbekzhan@gmail.com (B.Y.B.); 2 Central Research Institute of Epidemiology, 111123 Moscow, Russia; natalia-pshenichnaya@yandex.ru

**Keywords:** Crimean-Congo hemorrhagic fever, epidemiology, risk factors, Kazakhstan, tick-borne infections, natural focal diseases, Hyalomma ticks, vector surveillance, zoonotic diseases, One Health

## Abstract

Crimean–Congo hemorrhagic fever (CCHF) remains a major tick-borne zoonotic infection and a persistent public health challenge in Central Asia. Kazakhstan is among the endemic countries, where natural foci of CCHF are concentrated predominantly in the southern regions. This study evaluated the long-term epidemiological trends, regional distribution, seasonality, and risk factors associated with CCHF in Kazakhstan. A retrospective analysis of national surveillance data from 1980 to 2023 was conducted, complemented by detailed epidemiological and entomological investigations in the Turkestan region and the city of Shymkent during 2011–2023. Laboratory-confirmed cases, tick bite-related healthcare visits, entomological surveillance data, demographic characteristics, and epidemiological risk factors were analyzed. The results demonstrated cyclic fluctuations in CCHF incidence, with epidemic peaks occurring every 4–6 years and pronounced spring–summer seasonality, with the highest incidence observed in June and July. Long-term average annual incidence in endemic southern regions exceeded the national average by 2.67-fold. A total of 50,671 tick bite-related healthcare visits were registered during 2011–2023, while their incidence declined over time. Analysis of 94 laboratory-confirmed cases identified adults aged 18–40 years as the most affected group. Tick bites and livestock-related activities were the principal risk factors. These findings support the strengthening of integrated surveillance, vector control, laboratory diagnostics, and One Health-based prevention strategies in endemic regions.

## 1. Introduction

Crimean–Congo hemorrhagic fever (CCHF) is a severe zoonotic vector-borne viral disease caused by the Crimean–Congo hemorrhagic fever virus (CCHFV), a member of the genus *Orthonairovirus* within the family *Nairoviridae*. The disease is widely distributed across Africa, the Middle East, Asia, and southeastern Europe and represents one of the most important emerging tick-borne viral infections worldwide due to its epidemic potential, high case fatality rate, and expanding geographic distribution [1,2].

CCHFV is primarily transmitted by ixodid ticks of the genus *Hyalomma*, although infection may also occur through direct contact with the blood or biological fluids of infected humans and animals. Climatic changes, expansion of livestock farming, increasing human mobility, and environmental transformations contribute to the persistence and spread of natural CCHF foci in endemic regions. Due to its epidemic potential and public health significance, CCHF is included among priority pathogens requiring enhanced surveillance and preparedness strategies.

Kazakhstan is one of the CCHF-endemic countries in Central Asia. Natural foci of the infection are mainly concentrated in the southern regions of the country, including the Turkestan, Zhambyl, and Kyzylorda regions, where favorable climatic and ecological conditions support the circulation of *Hyalomma* ticks and the maintenance of the virus in natural ecosystems [3,4]. The Turkestan region and the city of Shymkent are characterized by intensive livestock farming, extensive rural populations, and environmental landscapes favorable for vector survival and reproduction.

Several studies have investigated different aspects of CCHF epidemiology and virology in Kazakhstan, including molecular characterization of circulating viral strains, seroepidemiological investigations, tick surveillance, and outbreak reports [5,6,7,8,9,10]. Previous studies demonstrated the circulation of multiple CCHFV genetic variants in southern Kazakhstan and confirmed the important role of *Hyalomma* ticks in maintaining endemic transmission [5,6]. In addition, recent investigations highlighted the influence of climatic and ecological factors on the spatial distribution of CCHF in Central Asia [9,10].

Despite these findings, comprehensive long-term analyses integrating epidemiological, entomological, demographic, and risk factor surveillance data across extended observation periods remain limited in Kazakhstan. In particular, insufficient attention has been paid to long-term epidemic cycles, the regional dynamics of tick bite incidence, associations between infected ticks and human morbidity, and the role of social and environmental determinants in sustaining endemic transmission.

Therefore, the present study aimed to analyze the long-term epidemiological trends, seasonal and regional characteristics, and major epidemiological risk factors of CCHF in Kazakhstan between 1980 and 2023, with detailed epidemiological and entomological assessment of the Turkestan region and the city of Shymkent between 2011 and 2023. The study also evaluated tick bite incidence, the distribution of laboratory-confirmed cases, and the associations between environmental, occupational, and demographic factors and CCHF morbidity in endemic regions of Kazakhstan.

## 2. Materials and Methods

A retrospective observational epidemiological study with elements of post hoc analysis was conducted to evaluate the long-term epidemiological trends and risk factors of Crimean–Congo hemorrhagic fever (CCHF) in the Republic of Kazakhstan between 1980 and 2023. In addition, an in-depth epidemiological and entomological analysis was performed in the Turkestan region and the city of Shymkent during the period 2011–2023, which represent the main endemic territories for CCHF in southern Kazakhstan.

The southern regions of Kazakhstan are characterized by a sharply continental climate with long hot summers and short, mild winters, creating favorable environmental conditions for the circulation of ixodid ticks of the genus *Hyalomma*, which are the principal vectors and reservoirs of CCHFV. The landscape of the region includes steppe, semi-desert, foothill, and river meadow ecosystems that support persistent natural foci of CCHF.

### 2.1. Data Sources

This study was based on official epidemiological surveillance data obtained from annual reports of the sanitary–epidemiological service of the Republic of Kazakhstan, including regional surveillance reports and annual reports of the Chief State Sanitary Officer of Kazakhstan. Additional data were obtained from regional sanitary–epidemiological control departments, laboratory surveillance databases, and entomological monitoring reports.

Data included annual incidence rates of CCHF; laboratory-confirmed and probable CCHF cases; demographic characteristics of patients; epidemiological risk factors; outpatient visits associated with tick bites; and entomological surveillance findings, including the proportion of CCHFV-positive ticks.

### 2.2. Case Definitions

Definitions of confirmed, probable, and suspected CCHF cases were based on the recommendations of the United States Centers for Disease Control and Prevention (CDC) and the national clinical protocol of the Republic of Kazakhstan.

A confirmed case was defined as a clinically compatible illness with laboratory confirmation of CCHFV infection by polymerase chain reaction (PCR), enzyme-linked immunosorbent assay (ELISA) detecting specific IgM/IgG antibodies, or other approved laboratory methods.

Probable and suspected cases were defined according to standard epidemiological and clinical criteria established in national surveillance guidelines.

### 2.3. Laboratory Confirmation

Laboratory confirmation of CCHF cases was performed at the Laboratory of Especially Dangerous Infections of the sanitary–epidemiological expertise service. Since 2009, laboratory diagnosis has included molecular detection of CCHFV RNA using PCR and serological testing using ELISA for specific IgM and IgG antibodies. Prior to 2009, serological diagnosis was primarily performed using indirect hemagglutination assays (IHA).

Commercial diagnostic kits manufactured by Vector-Best, Novosibirsk, Russia, were used for serological and molecular investigations according to the manufacturer’s instructions.

In fatal cases, confirmatory testing was additionally performed by the National Reference Laboratory of Kazakhstan.

### 2.4. Entomological Surveillance

Entomological monitoring and tick collection were carried out by the sanitary–epidemiological service in collaboration with the Shymkent Anti-Plague Station. Tick surveillance was conducted in endemic districts of the Turkestan region and the city of Shymkent.

Collected ticks were taxonomically identified, and testing for CCHFV was performed using standard laboratory diagnostic methods. Surveillance data included the number of collected ticks, the proportion of infected ticks, and the geographic distribution of positive samples.

### 2.5. Statistical Analysis

Statistical analysis was performed using Statistica version 10 (StatSoft Inc., Tulsa, OK, USA). Descriptive epidemiological methods were applied to assess incidence dynamics, seasonal patterns, regional distribution, and population risk groups.

Incidence rates were calculated per 100,000 population. Long-term trends were evaluated using linear regression analysis. Correlation analysis was performed to assess the relationships between CCHF incidence, tick bite incidence, and the proportion of infected ticks.

Risk ratios (RR) with 95% confidence intervals (CI) were calculated to evaluate epidemiological risk factors. Fisher’s exact test and chi-square tests were used for categorical variables where appropriate. Differences were considered statistically significant at *p* < 0.05.

### 2.6. Ethical Considerations

This study was approved by the local Ethics Committee of the South Kazakhstan Medical Academy (Protocol No. 12, dated 14 September 2022). The investigation used anonymized retrospective epidemiological surveillance data and did not involve direct intervention with human participants. All procedures were performed in accordance with national ethical standards and the principles of the Declaration of Helsinki.

## 3. Results

The Turkestan region and the city of Shymkent represent the principal CCHF-endemic territories in Kazakhstan due to favorable ecological and climatic conditions supporting persistent circulation of CCHFV-infected *Hyalomma* ticks [3,4].

Seasonal increases in CCHF transmission are observed annually in the region. Over the last 15 years, 110 CCHF cases have been registered in the Turkestan region, reflecting the resurgence of activity in natural CCHF foci. The interruption of systematic acaricide-based preventive measures, including cattle dipping and the treatment of livestock premises and pastures, was followed by an increase in the abundance of Hyalomma asiaticum and Dermacentor daghestanicus ticks and renewed activity of natural CCHF foci. The climate, expansion of livestock farming, and uncontrolled movement of livestock may also have contributed to this process [5].

Based on the results of the 2020 population census, more than 4.3 million people live in the southern regions of Kazakhstan. A large proportion of the population lives in the countryside. The leading economic sectors are agriculture, particularly farming and range sheep production, and extracting and manufacturing industries. The regional economy is predominantly based on agriculture and livestock farming, resulting in frequent human exposure to natural CCHF foci.

**Figure 1:**
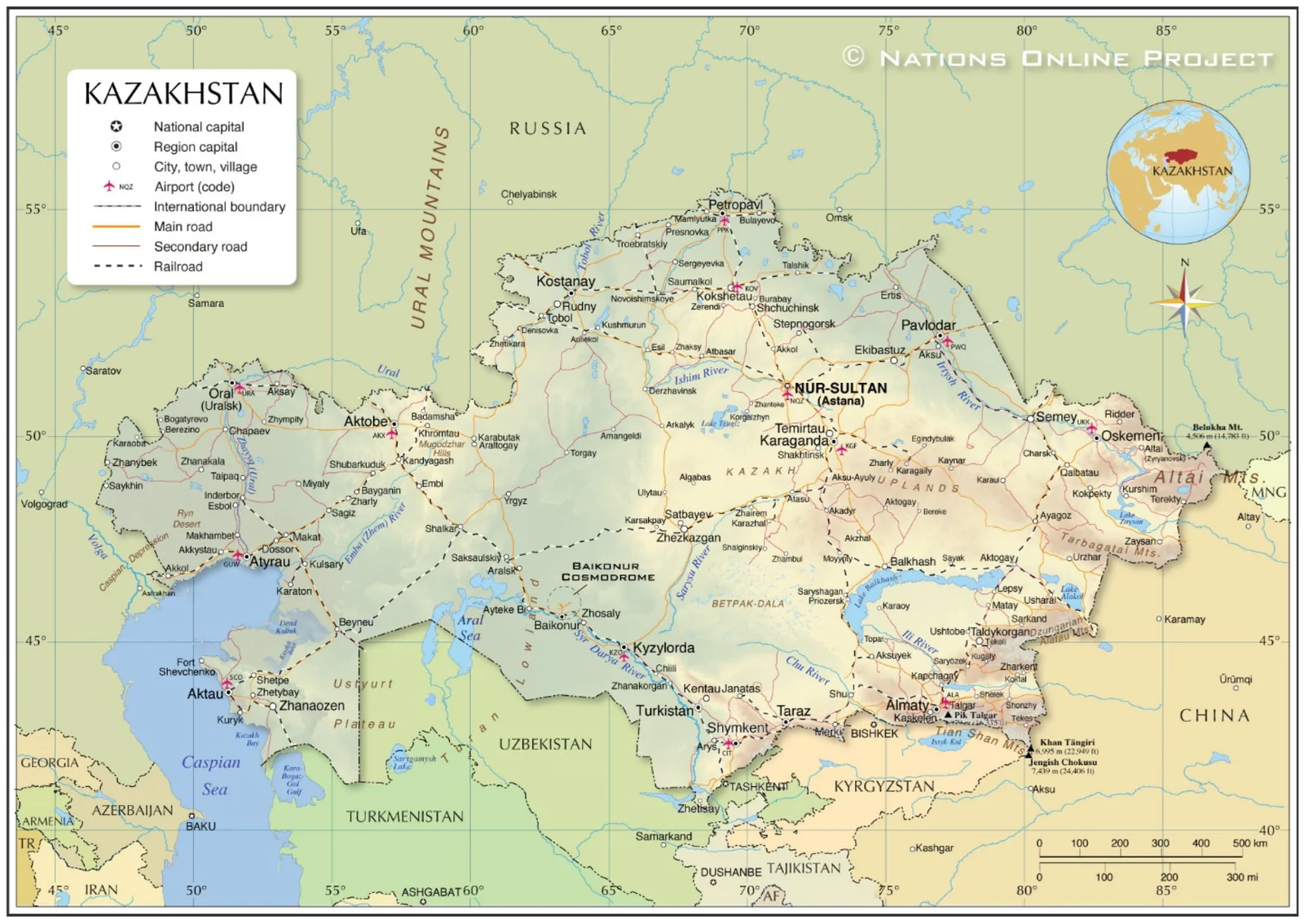
Geographic location of the Turkestan region and the city of Shymkent within the Republic of Kazakhstan. The highlighted territories represent the principal endemic regions for Crimean–Congo hemorrhagic fever (CCHF) characterized by persistent circulation of *Hyalomma* ticks and stable natural foci of CCHFV infection [6,7].

Southern Kazakhstan, including the Turkestan region and the city of Shymkent, has environmental characteristics that favor the persistence of zoonotic and tick-borne infections. The region has a sharply continental climate with long, hot summers and short, relatively mild winters, which extends the seasonal period of tick activity. Its steppe, semi-desert, foothill, and river–meadow landscapes provide diverse habitats for wildlife, livestock, and ixodid ticks. River valleys, irrigation systems, and reservoirs create localized humid habitats that may support tick survival. Extensive livestock farming increases contact among humans, domestic animals, and tick-infested environments. Natural CCHF foci in Kazakhstan are therefore concentrated mainly in steppe, semi-desert, forest–steppe, and river–meadow landscapes with relatively warm climates. These territories are widely used for livestock grazing and support the maintenance of Hyalomma ticks. The geographical location of the study area is presented in Figure 1.

There is a natural focus of CCHF on the territory in southern Kazakhstan, where the main vector and reservoir of the virus are ixodid ticks of the genus Hyalomma. CCHFV habitat practically coincides with the habitat of the key vector and reservoir (the ticks of the Hyalomma genus) and occupies a vast territory in the south of Kazakhstan.

The territory that is naturally focal to CCHF is confined to the steppe and semi-desert landscapes of the south of Kazakhstan. The intra-annual dynamics of the CCHF incidence rate are characterized by spring–summer seasonality. The first episodes of the infection are registered in March, reaching a peak in July, which is considered the period of the tick’s most aggressive attacking behavior. The last cases are registered in November.

**Figure 2:**
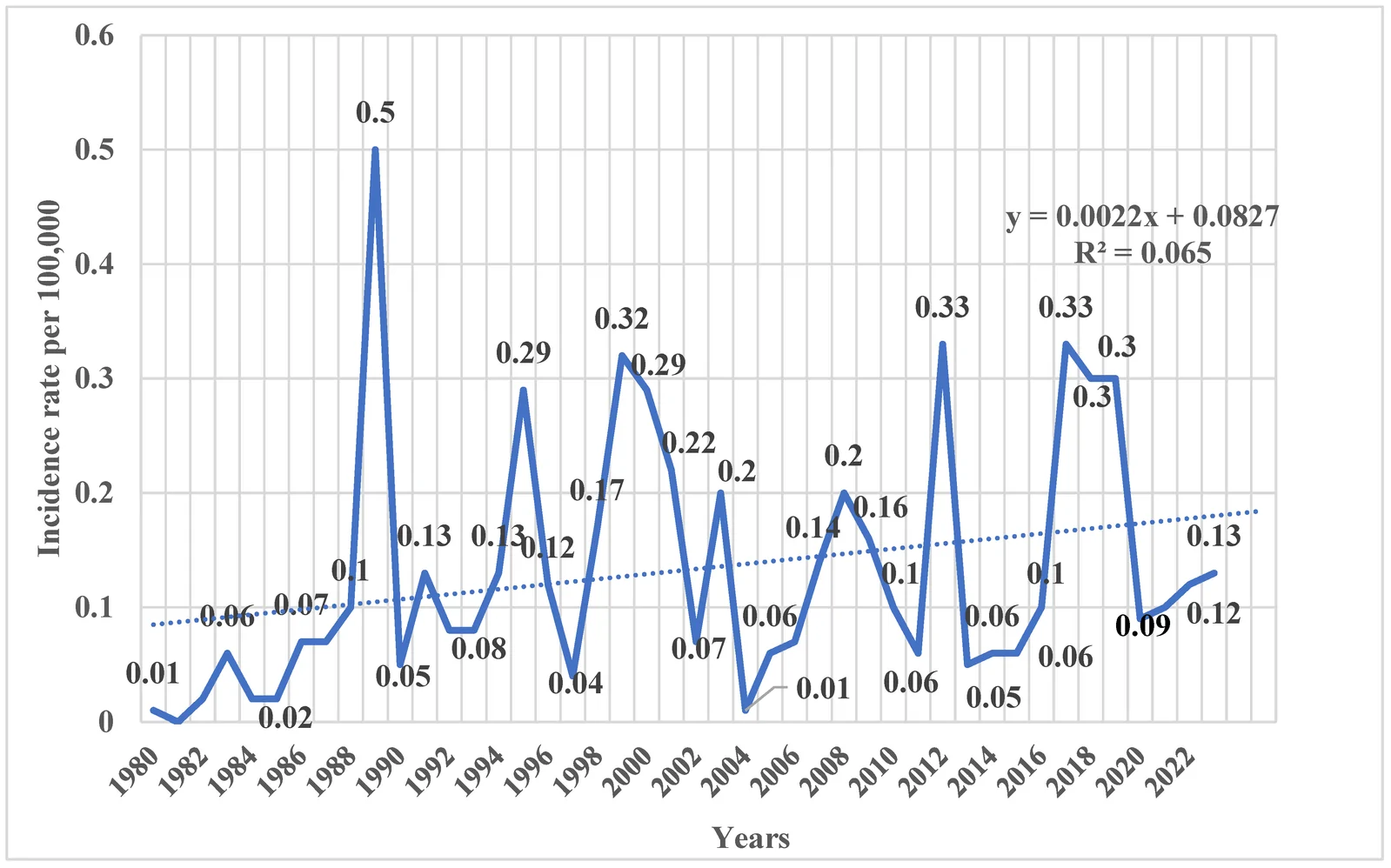
Long-term dynamics of Crimean–Congo hemorrhagic fever incidence in Kazakhstan between 1980 and 2023 (per 100,000 population). The graph demonstrates cyclic fluctuations in CCHF incidence, with epidemic peaks occurring approximately every 4–6 years. The dashed line represents the linear trend.

The CCHF incidence rate in Kazakhstan fluctuated from 0.010 ± 0.002 cases per 100,000 population in 1980 to 0.50 ± 0.20 cases per 100,000 population in 1989 (Figure 2) [8,9]. The long-term mean annual incidence was 0.130 ± 0.017 cases per 100,000 population. Incidence peaks were observed approximately every 4–6 years (1989, 1995, 1999, 2003, 2007, 2012, 2017, and 2023). However, annual quantitative data on Hyalomma tick density were not consistently available throughout 1980–2023; therefore, the observed periodicity cannot be directly attributed to fluctuations in tick population density. Linear regression demonstrated a slight long-term increase (y = 0.0022x + 0.0827; R^2^ = 0.065; annual rate of change = 6.5%), which may partly reflect improvements in laboratory diagnosis and surveillance rather than a true increase in transmission.

The key feature of CCHF and any other natural focal infection is its connection to landscapes with favorable environmental conditions for the circulation of the infectious agent. The foci of this disease are principally confined to semi-desert and steppe landscapes with a warm climate. The epidemic process is manifested in sporadic morbidity and outbreaks. The Turkestan region and the city of Shymkent are among the three CCHF-endemic zones in Kazakhstan, together with the Zhambyl and Kyzylorda regions, which correspond to the above- mentioned landscape types.

The CCHF long-term average annual incidence rate in the Turkestan region and the city of Shymkent between 2011 to 2023 exceeded the morbidity rate in Kazakhstan on the whole by 2.67 times (*p* < 0.01) (Table 1).

**Figure 3:**
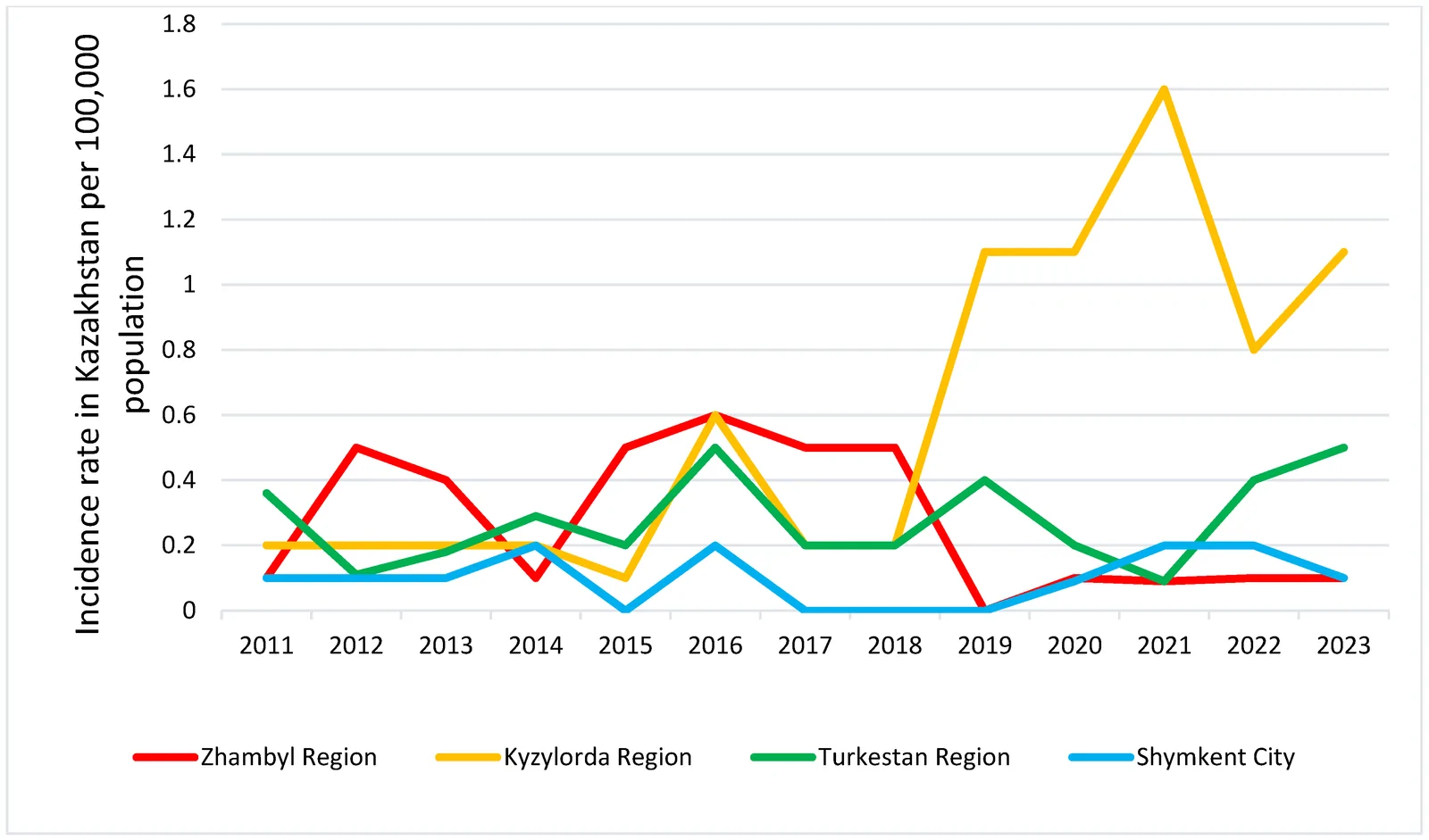
Dynamics of CCHF incidence across endemic regions of Kazakhstan between 2011 and 2023 (per 100,000 population), including the Turkestan region, Zhambyl region, Kyzylorda region, and the city of Shymkent.

Among the three endemic regions, the average CCHF incidence in the Turkestan region and the city of Shymkent was comparable to that in the Zhambyl region (0.3 per 100,000 population) and approximately twice that in the Kyzylorda region (*p* < 0.01) (Figure 3; Table 1). The Turkestan and Zhambyl regions are predominantly characterized by semi-steppe landscapes, whereas semi-desert landscapes are more common in Kyzylorda. Because this study did not include standardized inter-regional measurements of temperature, humidity, vegetation, or annual tick density, these ecological differences should be interpreted as descriptive contextual observations rather than quantified explanations for regional incidence differences.

**Figure 4:**
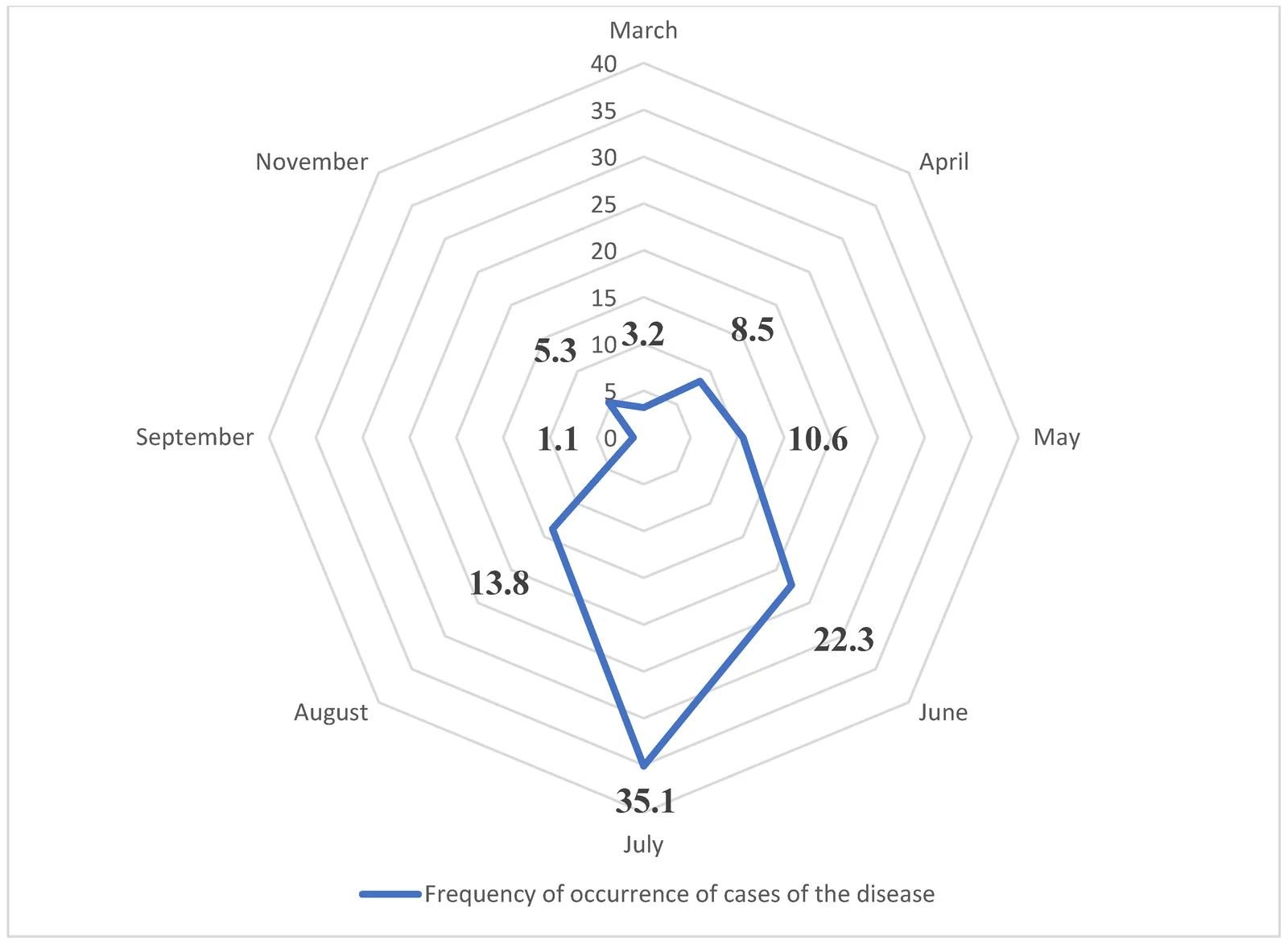
Seasonal distribution of laboratory-confirmed CCHF cases in Kazakhstan between 2011 and 2023. Peak incidence was observed during June and July, corresponding to the period of maximal seasonal activity of *Hyalomma* ticks.

Annually, the majority of CCHF cases were registered in July (35.1%), June (22.3%), and August (13.8%), connected with the increase in tick contacts due to both natural factors and human activities at this time (Figure 4).

The concentration of cases during the summer is consistent with increased opportunities for human–tick contact and seasonal agricultural and livestock-related activities. Direct annual measurements of Hyalomma population density were not available for the entire observation period.

One of the key indicators in the field of epidemiology is incidence of outpatient visits (i.e., the number of visits to healthcare institutions by the population) because of ixodid tick bites. These findings reflect both the epidemiologic and entomologic situation as they allow us to make judgements about the beginning and the end of the season when the natural focus is active. In addition, they give us an idea of the periods when the ticks are most numerous, and reveal the vectors’ spatial distribution in a designated territory. Moreover, these figures allow us to speak about the level of contact between ticks and humans, as well as about the effectiveness of the preventive activities.

A total of 50,671 people who suffered from tick bites were registered across the Turkestan region and the city of Shymkent between 2011 and 2023 (13 years), 37,894 of whom were reported in the Turkestan region. The regions with the highest incidence of outpatient visits for the whole period are as follows: the Baydibik district, which constituted 703.3 (maximum) visits per 100,000 population in 2011; the Ordabasy region, which made up 578.5 (maximum) visits per 100,000 population in 2019; and the Sayram district, which accounted for 360.9 (maximum) visits in 2011, which was probably connected to the increased grazing in these regions. However, there was a downward trend in incidence of outpatient visits connected with tick bites for the research period in the Turkestan region (*y* = −14.445 × *x* + 244.3), with the annual R_increase_ = −7.17%.

**Figure 5:**
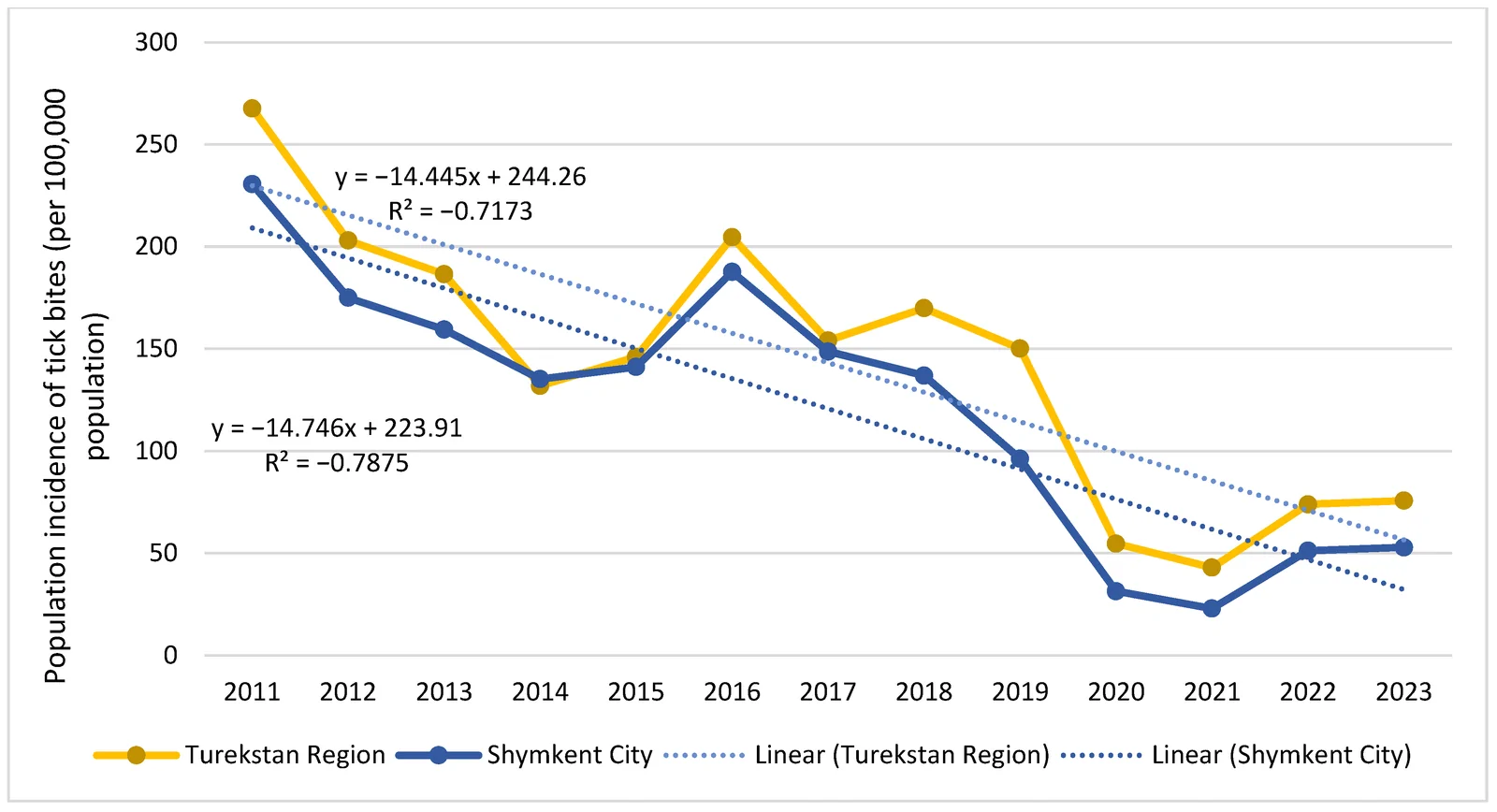
Dynamics of outpatient visits related to tick bites in the Turkestan region and the city of Shymkent between 2011 and 2023 (per 100,000 population). A gradual decrease in tick bite-related outpatient visits was observed during the study period. This ecological trend coincided with the expansion of acaricidal interventions and public health education, but the available data do not permit a causal assessment of their effectiveness.

A similar downward trend was observed in Shymkent during the same period (y = −14.746x + 223.91; R^2^ = 0.787), corresponding to an estimated annual decrease of 7.87% in tick bite-related outpatient visits (Figure 5).

Overall, tick bite-related outpatient visits decreased 3.4-fold in the Turkestan region (*p* < 0.01) and 2.3-fold in Shymkent (*p* < 0.05) during 2011–2023. Preventive activities during this period included annual acaricidal treatment of livestock, livestock premises, and selected pasture areas, together with public health education. The treated area was notably expanded in 2017–2018. However, standardized annual data on intervention coverage, frequency, and population reach were not consistently available; therefore, the observed decline should be interpreted as temporally associated with, rather than caused by, these measures.

Quantitative evaluation of the independent effect of individual preventive interventions was beyond the scope of the available surveillance dataset.

**Figure 6:**
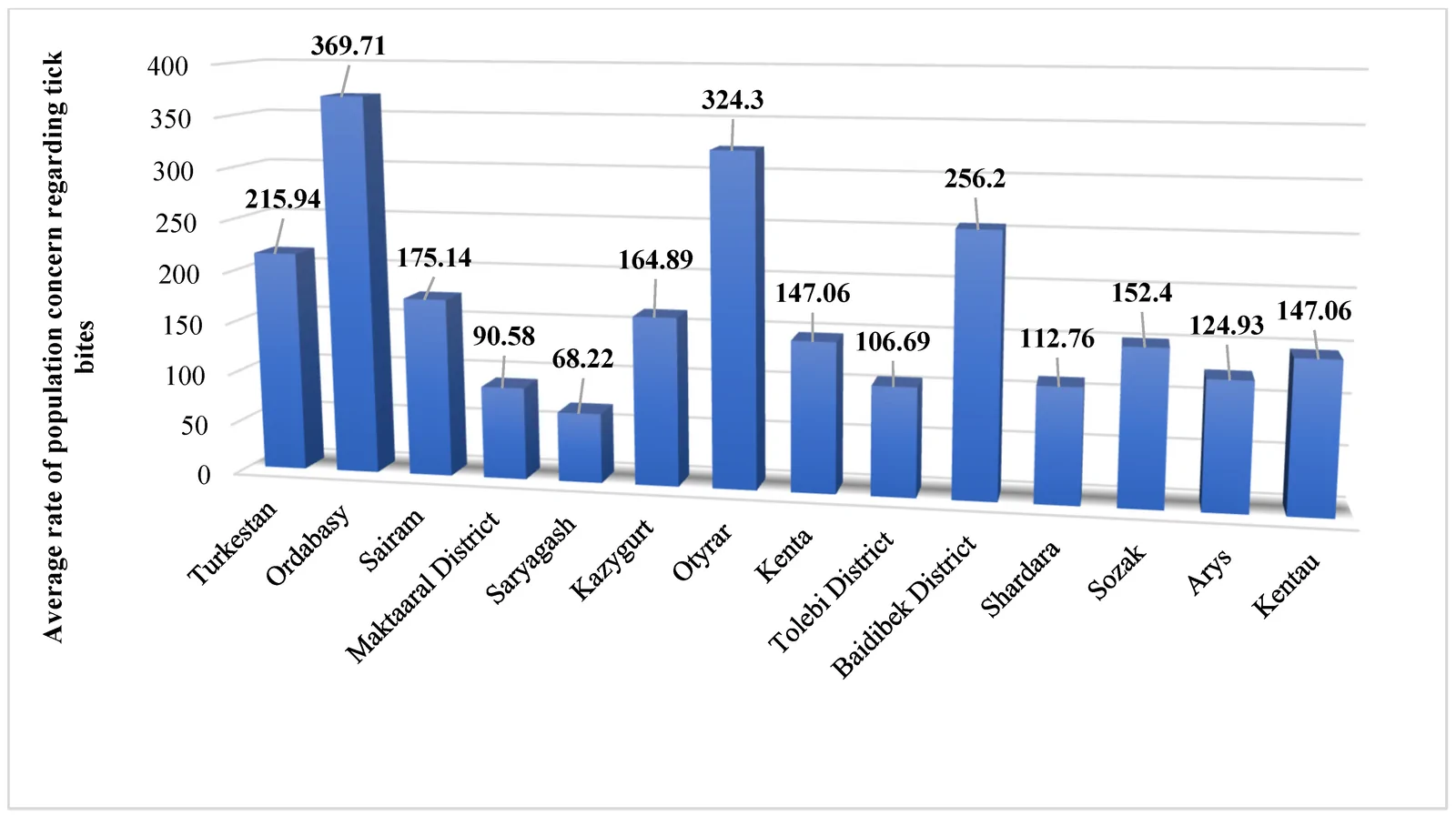
Average incidence of outpatient visits due to tick bites across the territories of the Turkestan region between 2011 and 2023.

Geographically speaking, the following districts prevail over others in terms of the incidence of outpatient visits due to tick bites: the incidence of outpatient visits made up 369.7 per 100,000 population in the Ordabasy district, constituted 324.3 per 100,000 population in the Otyrar district, and accounted for 256.2 per 100,000 population in the Baydibek district (Figure 6). This speaks to the territorial maldistribution of ticks due to landscape features and the activities with which the population is engaged, and it can reflect the effectiveness of preventive measures.

**Figure 7:**
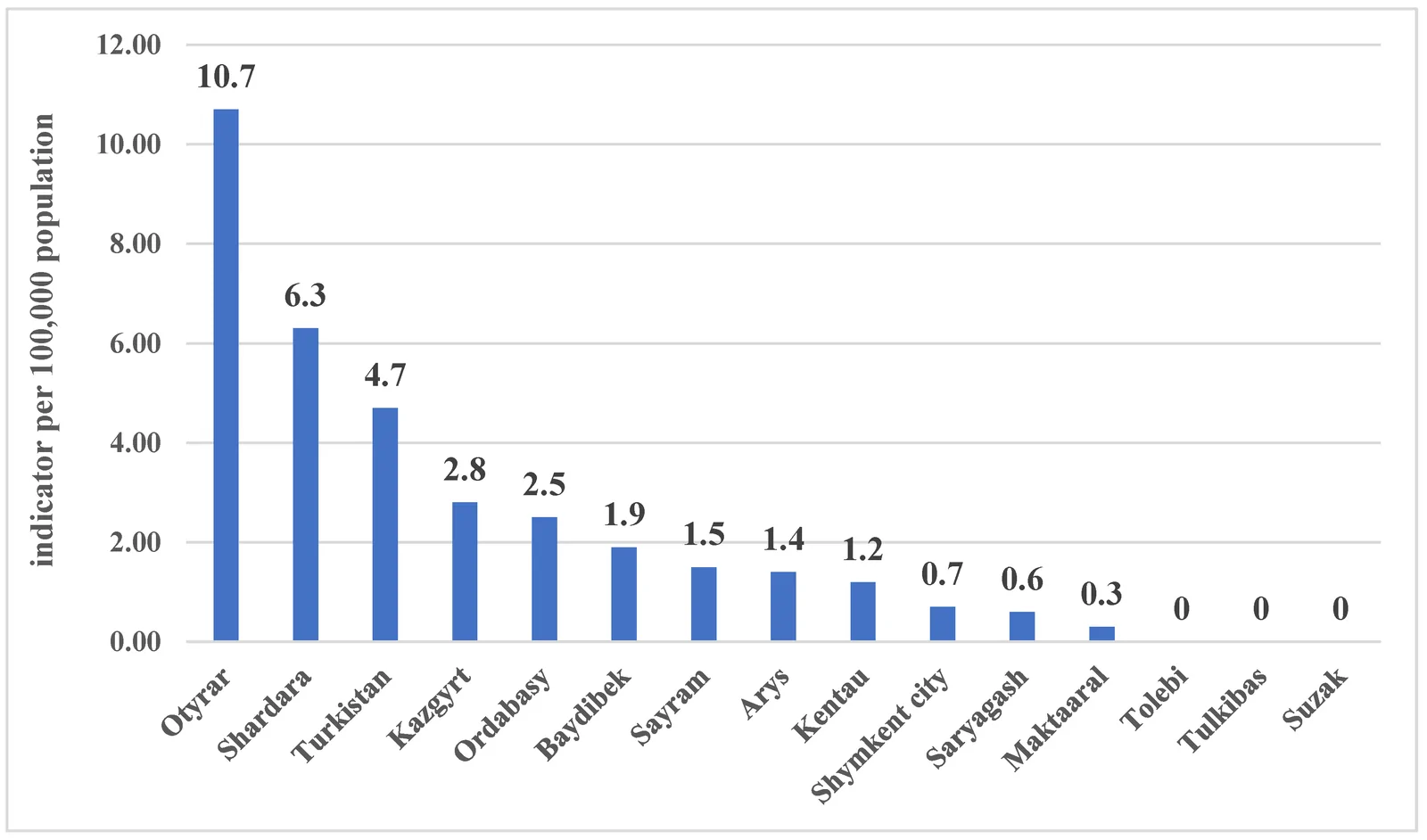
Total incidence rate of confirmed CCHF cases in different districts of the Turkestan region between 2011 and 2023.

Laboratory-confirmed cases of CCHF were registered in 12 (80%) districts of the Turkestan region between 2011 and 2023. Over the same span of time, the incidence rate in the population fluctuated significantly in and around the region, ranging from isolated cases to 10.7 per 100,000 population in the Otyrar district, 6.3 per 100,000 population in the Shardara district, and 4.7 per 100,000 population in the city of Turkestan. These findings indicate substantial geographic heterogeneity in CCHF transmission intensity across endemic districts and confirm the connection of the natural CCHF foci to designated territories and their expansion. There have been no CCHF cases registered in three districts within the Turkestan region, namely, Tolebi, Tulkibas, and Syzyq (Figure 7).

**Figure 8:**
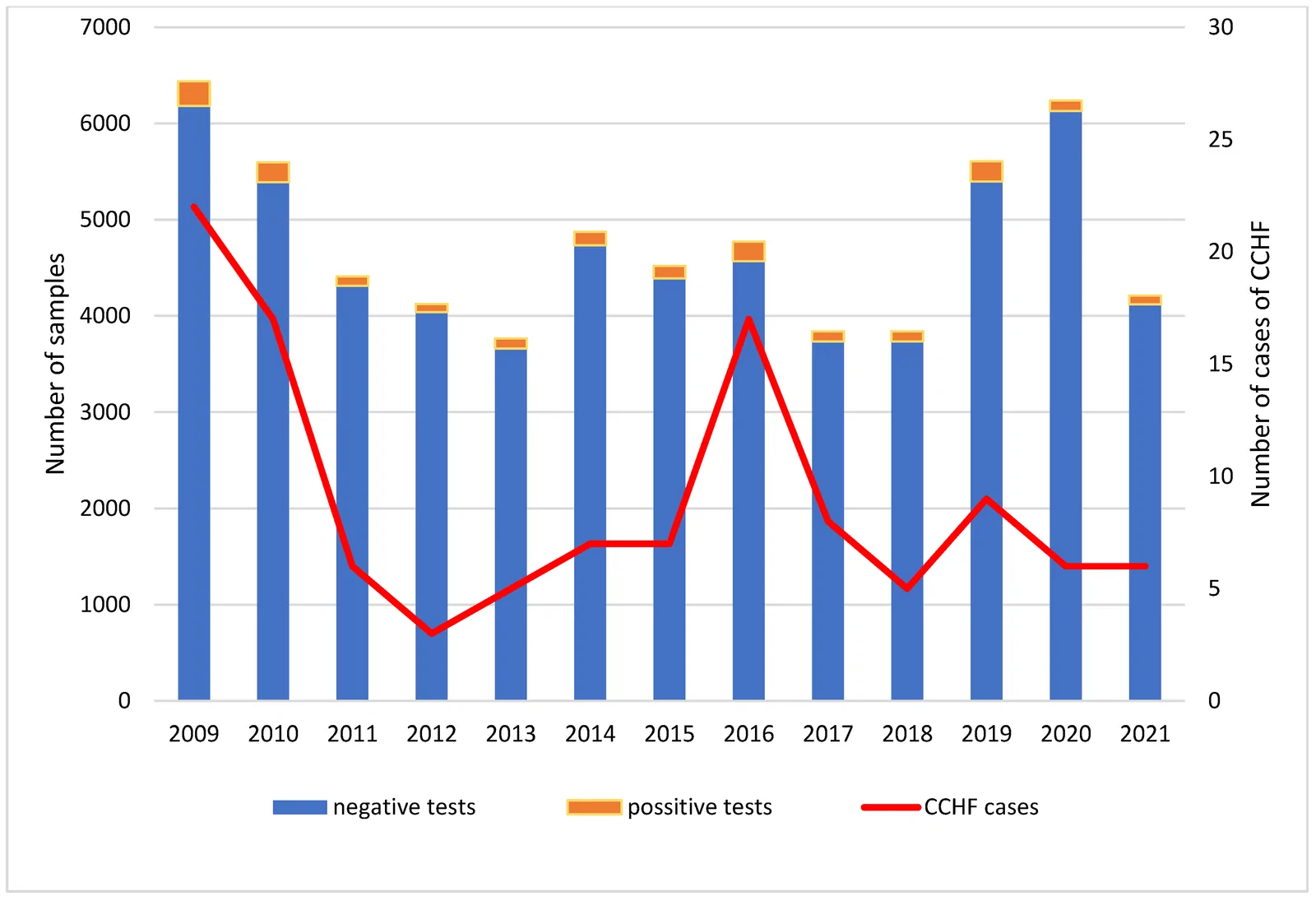
Dynamics of CCHF incidence rate in the population (laboratory-confirmed cases): total number of study samples along with the number of CCHF-positive study samples in the territory of the Turkestan region between 2009 and 2021.

**Figure 9:**
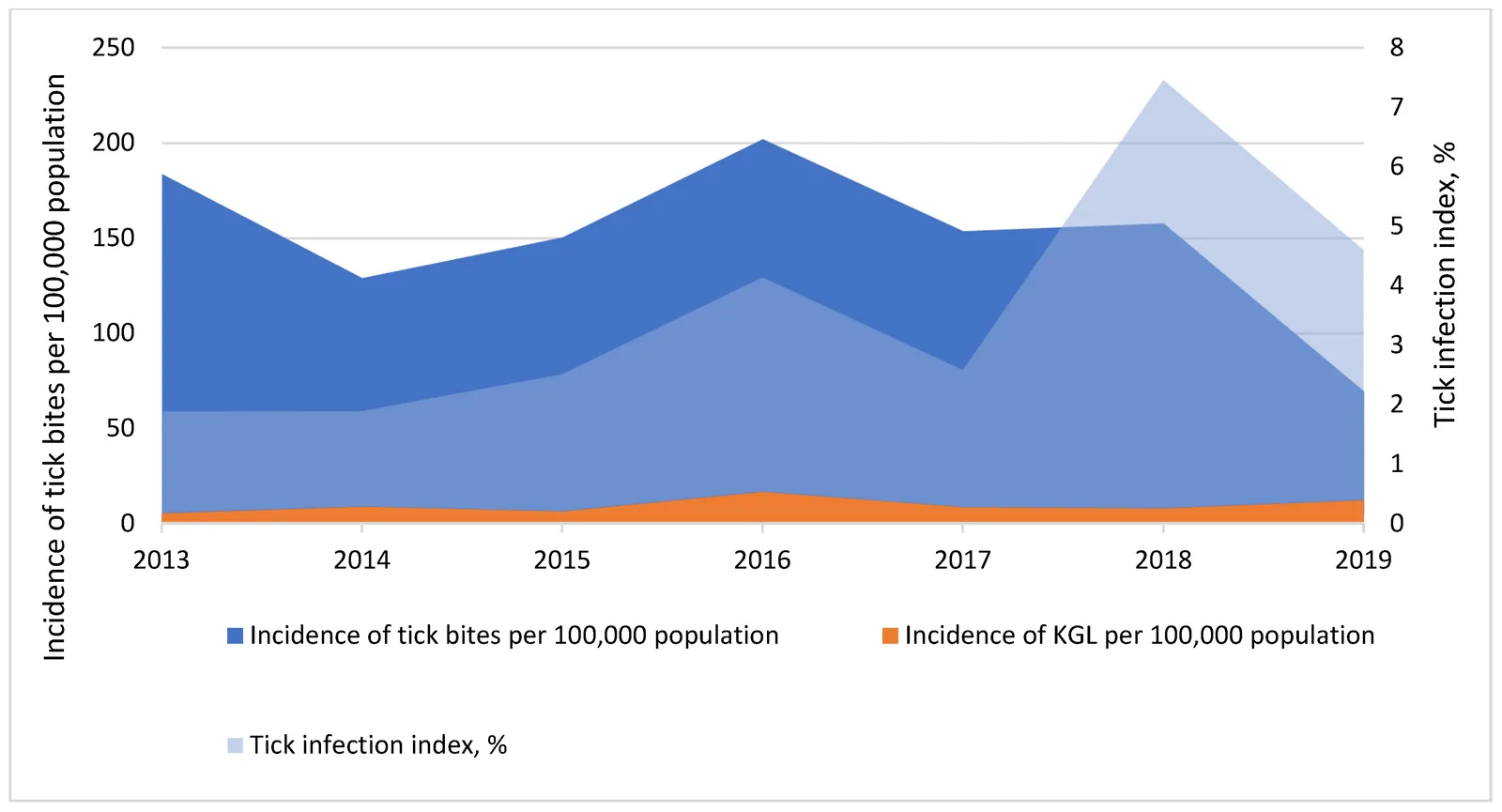
Long-term dynamics of CCHF incidence rate in the population due to tick bites (per 100,000 population): CCHF incidence rate per 100,000 population along with the index of CCHF-infected ticks in the territory of the Turkestan region and the city of Shymkent.

A study of the CCHF incidence rate in the population took into consideration the laboratory-confirmed cases, the total number of study samples, and the number of CCHF-positive ticks across the territory of the Turkestan region between 2009 and 2021. As the number of CCHF-positive ticks increased, an upward trend was recorded in CCHF cases across the population and vice versa; i.e., when the number of CCHF-positive ticks decreased, there was registered a downward trend in CCHF cases in the population (Figure 8 and Figure 9).

During 2017–2018, the surveillance reports documented an expansion of territories treated with acaricides. This expansion coincided with an increase in the proportion of CCHFV-positive ticks without a corresponding increase in tick bite-related outpatient visits or human incidence. Because detailed standardized coverage data were unavailable, this observation should be regarded as descriptive, and it does not establish the effectiveness of acaricidal treatment. The correlation between CCHF incidence and the proportion of CCHFV-positive ticks was moderate but not statistically significant (r = 0.42, *p* = 0.12).

The study of the dynamics of the CCHF incidence rate in the population in the territory of the Republic of Kazakhstan is key to understanding the epidemiologic situation and taking effective steps towards CCHF prevention and control. Analysis of the incidence rate dynamics allows us to point out the periods of increased virus activity, revealing its seasonal fluctuations, and to detect possible infection outbreaks. However, in order to obtain a clear vision of the reasons for these changes and the development of effective preventive measures, it is crucial to consider the population groups who run a greater risk of being CCHF-infected, along with the key risk factors influencing CCHF incidence rate.

The identified epidemiological risk factors may be categorized into the ecological, social, and behavioral determinants of transmission. The ecologic factors comprise the climatic conditions contributing to vectors’ activity and reproduction, along with the presence of natural CCHF foci. The social factors include the level of medical service, hygienic and sanitary conditions, and population density in endemic regions. The behavioral factors include the actions and habits of individual people, such as the individual protection measures taken while working outdoors and one’s propensity to immediately seek health advice with the onset of symptoms.

Population groups that are at risk of being infected with CCHF include agricultural workers and people living in endemic regions, as well as those who have a direct contact with infected animals and natural virus foci.

### Risk Factors of CCHF Contamination Among Different Population Groups in Kazakhstan

**Figure 10:**
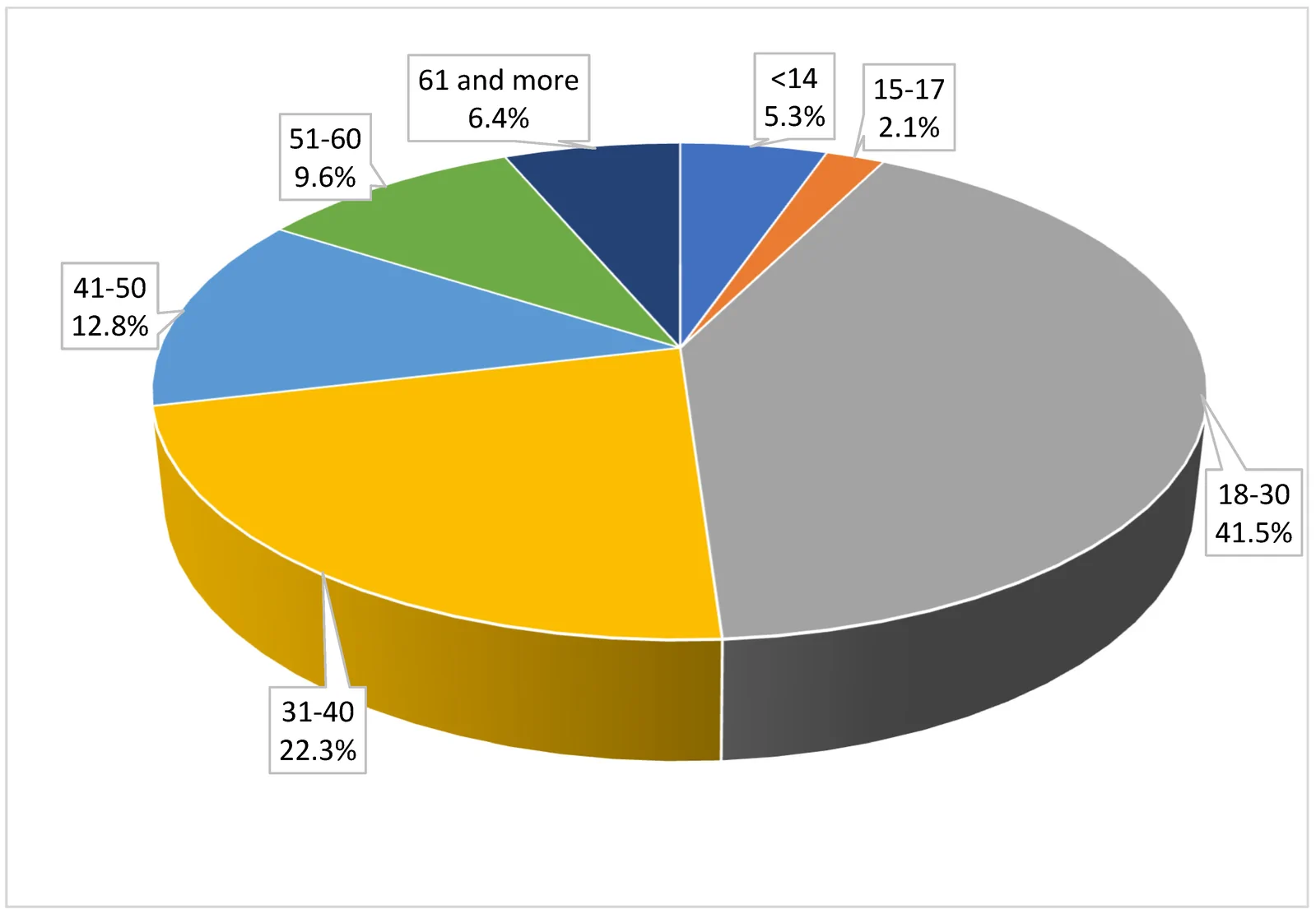
Age pattern of people suffering from CCHF (*n* = 94).

Analysis of cases of CCHF incidence revealed differences across age groups (Figure 10). The population groups aged between 18 and 30 (41.5%) and 31 and 40 (22.3%) hold the highest ratio. This fact can be explained by the more frequent incidences of contact within these population groups and the natural CCHF foci.

Children aged between 0 and 14 (5.3%) and the population group aged between 15 and 17 (2.1%) have an insignificant ratio with respect to the total CCHF incidence rate. The average age of people suffering from CCHF came to 42.0 ± 1.8 years, out of which 84.0% were at the age of 50.

**Figure 11:**
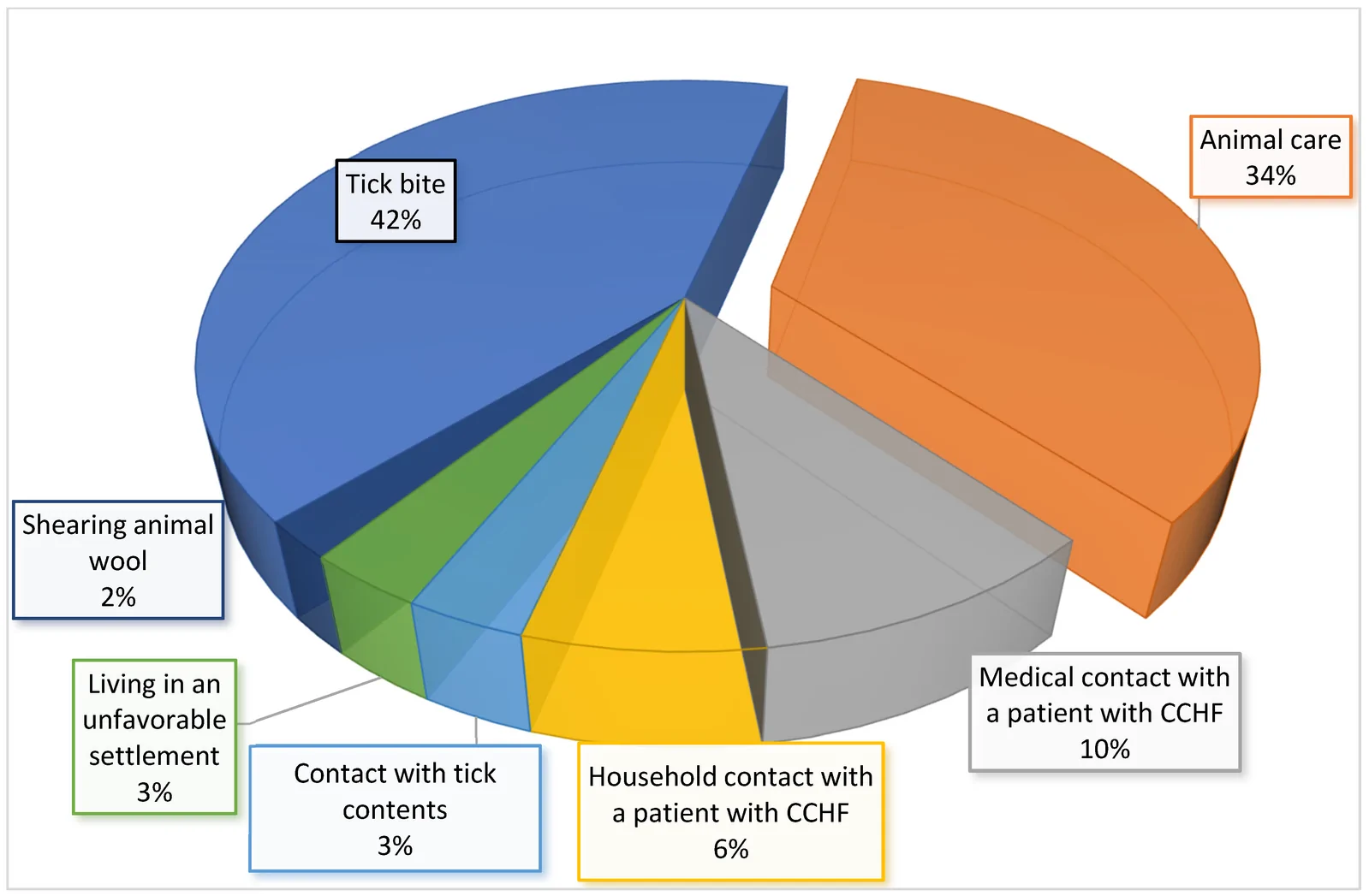
Risk factors in epidemiologic anamnesis of CCHF-infected patients (*n* = 94).

The most frequent epidemiologic risk factor was a tick bite, indicated by 41.5% of the patients. A total of 34% of patients were looking after livestock animals; 9.6% of patients reported in-hospital contact with CCHFV; 3.2% of patients were exposed to a tick’s fluid while removing it from domestic animals, clothes, etc.; no epidemiologic factors except for living in an endemic region were recorded for 3.2% of the patients; and 2.1% of patients shore sheep (Figure 11).

**Figure 12:**
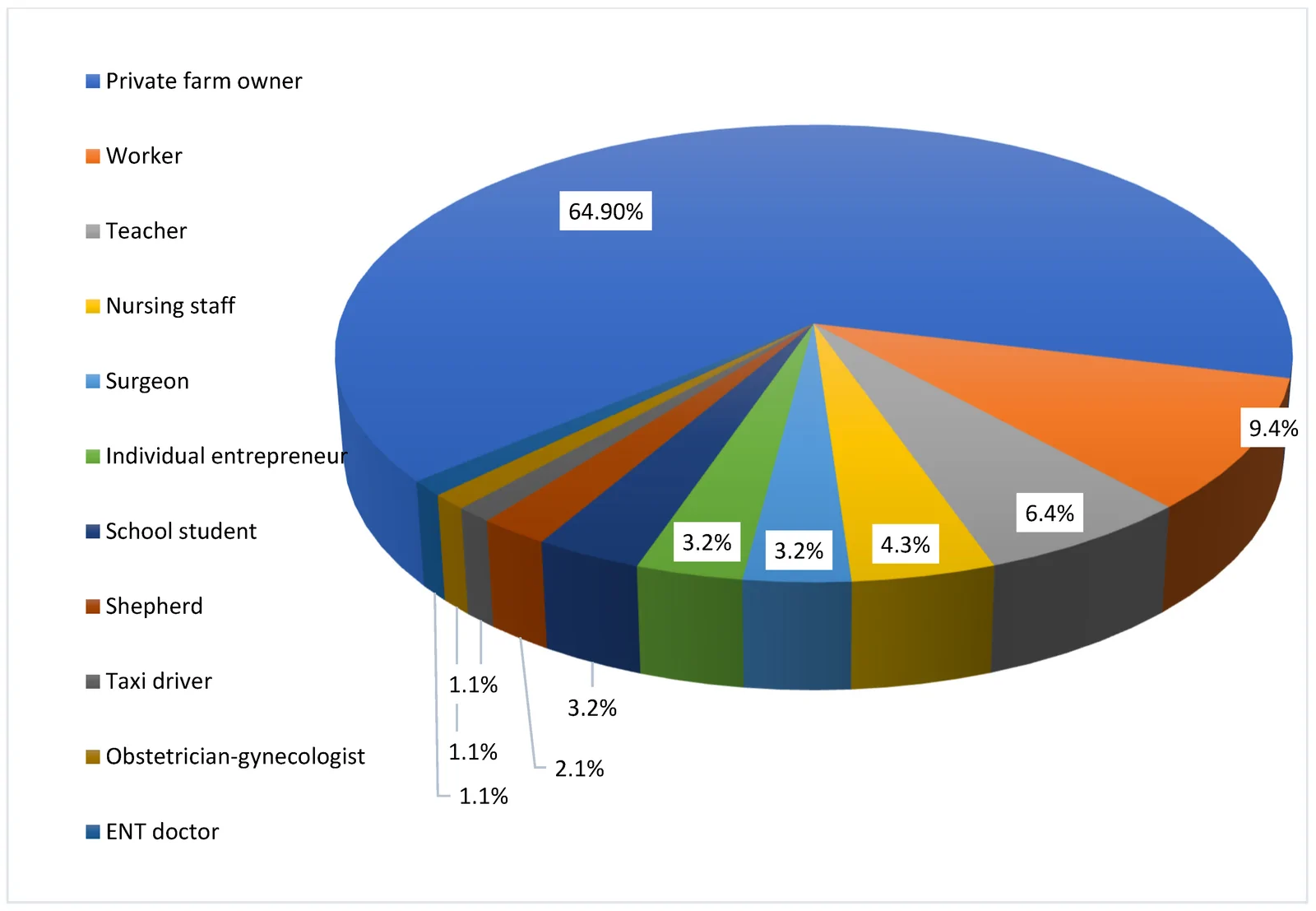
CCHF incidence pattern in different social and occupational population groups (*n* = 94).

The low percentage of tick bites can be explained by the fact that normally, 30% of the patients cannot recall being bitten by a tick. Additionally, there could be other epidemiologic risk factors of disease development or there could be a set of several factors. In 87% of CCHF cases, the patients reported ownership of cattle in private households; they also indicated caring for free-range dogs and cats and managing small cattle and livestock (80% of cases). Concerning the CCHF incidence pattern across different social and occupational population groups, people with private farms (64.9%) were more often affected by CCHF infection; general workers laboring in natural infection foci were the second-most-affected group (9.4%); and on the whole, the number came to 9.7% for all categories of medical workers (i.e., nurses and surgeons, obstetrician–gynecologists, and otolaryngologists) (Figure 12).

We carried out a univariate analysis to determine the differences in risk factors between laboratory-confirmed CCHF cases and probable/assumptive cases, focusing on the year 2016, when the maximum number of CCHF cases (15) was registered during a season, and the number of assumptive/probable CCHF cases which were not laboratory-confirmed came to 33 and 37, respectively (*n* = 37) (Table 2).

Univariate analysis (Table 3) identified statistically significant associations with cattle ownership (RR = 6.5; *p* = 0.0007), small ruminant ownership (RR = 6.7; *p* = 0.00001), cattle care (RR = 5.0; *p* = 0.001), small ruminant care (RR = 3.8; *p* = 0.002), and dog care (RR = 4.0; *p* = 0.001). These estimates describe unadjusted associations. The dataset did not include standardized measures of exposure duration or sufficient covariate information for multivariable adjustment; therefore, residual confounding cannot be excluded, and the risk ratios should not be interpreted as independent causal effects.

There were no patients engaged in cattle slaughtering or quartering with a laboratory-confirmed CCHF result or suspicion of CCHF in the year 2016. The findings confirm the importance of particular risk factors in CCHF contamination and highlight the urgency of recommendations concerning the improvement of preventive measures in the use of personal protective equipment while slaughtering or cutting meat and managing livestock.

The research on the population groups and CCHF risk factors in the territory of the Republic of Kazakhstan is considered to be one part of the epidemiologic analysis and preventive measures taken to combat this infectious disease.

Risk factors such as climatic conditions, the density and activity of ticks, social and economic conditions, behavioral habits, and the capabilities of medical services have a significant impact on the spread of CCHF. Agricultural workers and people living in endemic regions, along with those who often visit natural infection foci, are the most vulnerable population groups. These factors create favorable conditions for virus circulation and contribute to the increase in incidence rate. The at-risk population groups and the various risk factors define the overall picture of the infection and allow us to predict possible outbreaks.

## 4. Discussion

The present study provides one of the most comprehensive long-term epidemiological analyses of Crimean–Congo hemorrhagic fever (CCHF) in Kazakhstan, covering more than four decades of surveillance data and integrating epidemiological, entomological, demographic, and risk factor assessments. The findings confirm that CCHF remains a significant public health concern in the southern regions of Kazakhstan and demonstrate the persistent activity of natural foci associated with *Hyalomma* ticks.

One of the key findings was the presence of long-term cyclic fluctuations in reported CCHF incidence, with peaks occurring approximately every 4–6 years. Similar patterns have been reported in other endemic settings and may be influenced by climatic variability, livestock movement, vector ecology, and changes in surveillance [10,11,12,13,14]. However, the present dataset did not contain continuous annual measurements of Hyalomma density for 1980–2023. Consequently, the observed periodicity cannot be reliably attributed to ecological fluctuations in tick population size and should be regarded as a descriptive epidemiological pattern.

The present study additionally demonstrated pronounced spring–summer seasonality of CCHF morbidity, with the highest number of cases occurring in June and July, corresponding to periods of maximal activity of *Hyalomma* ticks. Similar seasonal patterns have been described in Turkey, Iran, Afghanistan, and other endemic territories [11,12,13,14,15]. Increased agricultural activities and intensive human contact with livestock during these months may further contribute to transmission risk.

An important strength of this study is its combined assessment of tick bite-related outpatient visits, entomological surveillance, and laboratory-confirmed human cases. Tick bite-related visits may serve as an indirect indicator of human contact with active natural foci. The decline in these visits coincided with the expansion of acaricidal treatment, particularly in 2017–2018, and ongoing public health education. Nevertheless, because standardized annual data on intervention coverage and intensity were incomplete, this study cannot determine whether preventive measures caused the observed decline.

This study also revealed substantial regional heterogeneity in reported CCHF incidence. The Turkestan region and Shymkent had higher average incidence than the national population. These areas are characterized by extensive livestock farming and frequent human contact with natural foci. Although semi-steppe and irrigated landscapes may provide suitable habitats for Hyalomma ticks, this study did not compare temperature, humidity, vegetation, or tick density across regions. The contribution of specific environmental parameters to regional incidence differences therefore could not be quantified.

Adults aged 18–40 years represented the most affected group, consistent with their frequent participation in agricultural and livestock-related activities. Univariate analysis identified associations between confirmed CCHF and livestock ownership or care. However, exposure duration was not measured, and the analysis did not adjust for potential confounders such as occupation, place of residence, concurrent animal exposures, or use of personal protective measures. These findings should therefore be interpreted as hypothesis-generating associations rather than independent causal risk estimates.

The presence of healthcare-associated exposure among some patients additionally highlights the continuing importance of infection prevention and control measures in medical institutions. Nosocomial transmission of CCHFV remains a recognized problem in endemic regions, particularly in situations involving delayed diagnosis or insufficient adherence to biosafety precautions. Therefore, strengthening awareness among healthcare workers and improving early recognition of CCHF remain important components of regional preparedness strategies.

The obtained findings support the importance of the One Health approach in CCHF surveillance and prevention. Sustainable control of the disease requires coordinated interaction between epidemiological surveillance services, veterinary systems, entomological monitoring, environmental assessment, and healthcare institutions. Integrated surveillance systems may improve early detection of epidemiological changes and contribute to timely preventive interventions in endemic territories.

This study has several strengths. First, it includes one of the longest observation periods reported for CCHF epidemiology in Kazakhstan. Second, the study combines epidemiological surveillance, entomological monitoring, demographic analysis, and risk factor assessment. Third, the investigation is based on official national surveillance data and laboratory-confirmed cases obtained from endemic regions with persistent natural foci.

Several limitations should be acknowledged. First, the retrospective observational design may be affected by incomplete historical reporting and changes in surveillance intensity. Laboratory diagnostic methods evolved over time, with PCR and ELISA becoming routinely available only after 2009, and mild or asymptomatic infections may have remained undetected. Second, the entomological dataset included the number of collected and CCHFV-positive ticks but did not provide standardized annual Hyalomma density estimates throughout 1980–2023; thus, the 4–6-year incidence cycles cannot be directly linked to tick population dynamics. Third, detailed annual data on the timing, coverage, frequency, and intensity of acaricidal interventions and public education campaigns were incomplete, precluding causal evaluation of their effect on tick bite-related outpatient visits. Fourth, the risk factor analysis was univariate, did not quantify exposure duration, and could not fully control for confounding. Finally, standardized inter-regional data on temperature, humidity, vegetation, and other ecological parameters were unavailable, so the environmental contribution to regional differences in incidence could not be quantified.

Overall, the study demonstrates persistent endemic CCHF transmission in southern Kazakhstan. The findings support continued epidemiological and entomological surveillance, improved collection of standardized tick density and intervention-coverage data, strengthened laboratory diagnostics, and integrated One Health strategies. Prospective studies incorporating quantitative ecological measurements and multivariable risk factor analysis are needed to clarify the drivers of periodic incidence fluctuations and regional heterogeneity.

## 5. Conclusions

The present study demonstrates that CCHF remains an epidemiologically important zoonotic infection in southern Kazakhstan. Long-term surveillance identified recurrent incidence peaks approximately every 4–6 years and pronounced spring–summer seasonality. Because continuous annual tick density data were unavailable, the periodic peaks should not be interpreted as direct evidence of cyclic changes in Hyalomma population size.

The Turkestan region and Shymkent had higher average incidence than the national population. Livestock ownership and management, animal care, tick bites, and residence in endemic rural areas were associated with infection; however, these unadjusted associations may be affected by confounding. The contribution of specific environmental factors could not be quantified because comparable regional measurements of climate, vegetation, and tick density were not available.

The integrated analysis demonstrates the value of combining human surveillance, tick bite-related healthcare visits, and entomological testing. The temporal coexistence of declining tick bite-related visits and expanded preventive activities is noteworthy, but causal effectiveness could not be established from the available retrospective data.

To our knowledge, this study represents one of the longest integrated epidemiological and entomological analyses of CCHF conducted in Kazakhstan and Central Asia. The findings emphasize the necessity for continued strengthening of laboratory diagnostics, acaricidal interventions, epidemiological surveillance, healthcare worker preparedness, and public health education programs.

Overall, effective control of CCHF in endemic regions requires the implementation of coordinated One Health-oriented strategies integrating human, veterinary, entomological, and environmental surveillance systems to reduce transmission risks and improve regional epidemic preparedness.

## Figures and Tables

**Table 1:** CCHF incidence rate in Kazakhstan between 2011 and 2023 (in absolute numbers and per 100,000 population).

Territory	Indicator	Years													Absolute Number of Patients	Average Annual Indicator per 100,000 Population (per 100,000 Population)
		2011	2012	2013	2014	2015	2016	2017	2018	2019	2020	2021	2022	2023		
**Turkestan Region**	Absolute number of patients	10	3	5	8	6	15	8	5	9	5	4	12	13	103	
	Indicator per 100,000 population	0.36	0.11	0.18	0.29	0.21	0.54	0.28	0.26	0.4	0.2	0.19	0.59	0.63		0.32
**Republic of Kazakhstan**	Absolute number of patients	14	11	11	12	12	27	16	18	18	17	19	41	40	208	
	Indicator per 100,000 population	0.06	0.07	0.09	0.16	0.06	0.1	0.33	0.3	0.3	0.2	0.1	0.21	0.20		0.12

**Table 2:** Epidemiological risk factors among suspected, probable, and laboratory-confirmed CCHF cases in the Turkestan region in 2016.

Risk Factor	Presumed Cases (*n* = 33)		Probable Cases (*n* = 37)		Confirmed Cases (*n* = 15)	
	*n*	%	*n*	%	*n*	%
Animal care	12	36	10	27	12	80
Presence of cattle	11	33	18	49	13	87
Presence of small ruminants	6	18	14	38	12	80
Presence of dogs	5	15	2	5	9	60
Male gender	24	73	27	73	11	73
Cattle care	10	30	10	27	12	80
Small ruminant care	5	15	8	22	9	60
Dog care	3	9	3	8	8	53
Slaughter of animals	0	0	0	0	0	0
Tick bite	26	79	12	32	7	47
Meat cutting	0	0	0	0	0	0
Possible contact in hospital 2 weeks prior to illness	3	9	1	3	0	0

**Table 3:** Univariate analysis of epidemiological risk factors associated with laboratory-confirmed CCHF cases in the Turkestan region in 2016.

Risk Factor	Confirmed Cases (*n* = 15)	%	Risk Ratio	95% CI	*p* (Fisher’s Exact Test)
Presence of cattle	13	87	6.5	1.6–26.0	0.0007
Presence of small ruminants	12	80	6.7	2–20	0.00001
Dog care	8	53	4	2–7	0.001
Cattle care	12	80	5	1.6–16.0	0.001
Small ruminant care	9	60	3.8	1.7–8.8	0.002

## Data Availability

Data are available from the corresponding author upon reasonable request.
